# Supplementary material for: Cell-Free RNA from Plasma in Patients with Neuroblastoma: Exploring the Technical and Clinical Potential
Source: Cancers (Basel). 2023 Mar 31;15(7):2108. doi: 10.3390/cancers15072108 (PMC10093559; doi:10.3390/cancers15072108)
Supplement: Supplementary file 1 [file cancers-15-02108-s001.zip › cancers-2251851-supplementary.pdf]

Supplemental Table S1. Sequences of ddPCR assays.

**MCM2**	**Forward**	CACATCCATGTCCGCATCTC
	**Reverse**	GTTGCAGTTGTACTTGACCATGC
	**Probe**	/5HEX/ACTGGCGTC/ZEN/CTGCCCCAGCTC/3IABkFQ/
**CDC6**	**Forward**	CAAATTCTGAGCAGAGATGTCCACT
	**Reverse**	TGACATCCATCTCCCTTTCCC
	**Probe**	/56-FAM/CCCAGATCG/ZEN/GCTGCCTGCCA/3IABkFQ/
**H2AFZ**	**Forward**	GTTTCCCGCTCGCAGAGA
	**Reverse**	GTGAGGTACTCCAGGATGGCTG
	**Probe**	/56-FAM/CATGGACGT/ZEN/GTGGGCGCGACT/3IABkFQ/
**ATAD2**	**Forward**	CAACTTGCTAATGGCAGGCA
	**Reverse**	TTCTAGCCCTCAATGACCGAGTA
	**Probe**	/5HEX/AGCCTGCTG/ZEN/TCTGGCCAACTGCCT/3IABkFQ/
**E2F1**	**Forward**	CAGCTGGACCACCTGATGAATA
	**Reverse**	GGTCTGCAATGCTACGAAGGTC
	**Probe**	/56-FAM/CCTCGGAGA/ZEN/GCAGGCGCAGC/3IABkFQ/
**DHFR**	**Forward**	GGTTCGCTAAACTGCATCGTC
	**Reverse**	AGAGGTTGTGGTCATTCTCTGGA
	**Probe**	/56-FAM/CGGTGGCCA/ZEN/GGGCAGGTCC/3IABkFQ/
**PHOX2B**	**Forward**	GGCACCCTCAGGGACCA
	**Reverse**	CTGCGCGCTCCTGCTT
	**Probe**	/56-FAM/CCAGAACCG/ZEN/CCGCGCCAA/3IABkFQ/
**B2M**	**Forward**	GAGTATGCCTGCCGTGTG
	**Reverse**	AATCCAAATGCGGCATCT
	**Probe**	/5HEX/CCTCCATGA/ZEN/TGCTGCTTACATGTCTC/3IABkFQ/
**GUSB**	**Forward**	GAAAATATGTGGTTGGAGAGCTCATT
	**Reverse**	CCGAGTGAAGATCCCCTTTTTA
	**Probe**	/5HEX/CCAGCACTC/ZEN/TCGTCGGTGACTGTTCA/3IABkFQ/
**TH**	**Forward**	ATT GCT GAG ATC GCC TTC CA
	**Reverse**	AAT CTC CTC GGC GGT GTA CTC

	**Probe**	/56-FAM/ACA GGC ACG GCG/ZEN/ ACC CGA TTC /3IABkFQ/
**CHRNA3**	**Forward**	GTCCATGTCTCAGCTGGTGAAG
	**Reverse**	TTCCATTTCAGCTTGTAGTCATTCC
	**Probe**	/56-FAM/CAGATCATG/ZEN/GAGACCAACCTGTGGCTC/3IABkFQ/
**HPA1A/B**	**Forward**	CCAACATCTGTACCACGCGA
	**Reverse**	GGCACAGTTATCCTTCAG
	**HPA1A probe**	/56-FAM/CCTGCCTCT/ZEN/GGGCTCACCTC/3IABkFQ/
	**HPA1B probe**	/56-FAM/CCTGCCTCC/ZEN/GGGCTCACCTC/3IABkFQ/

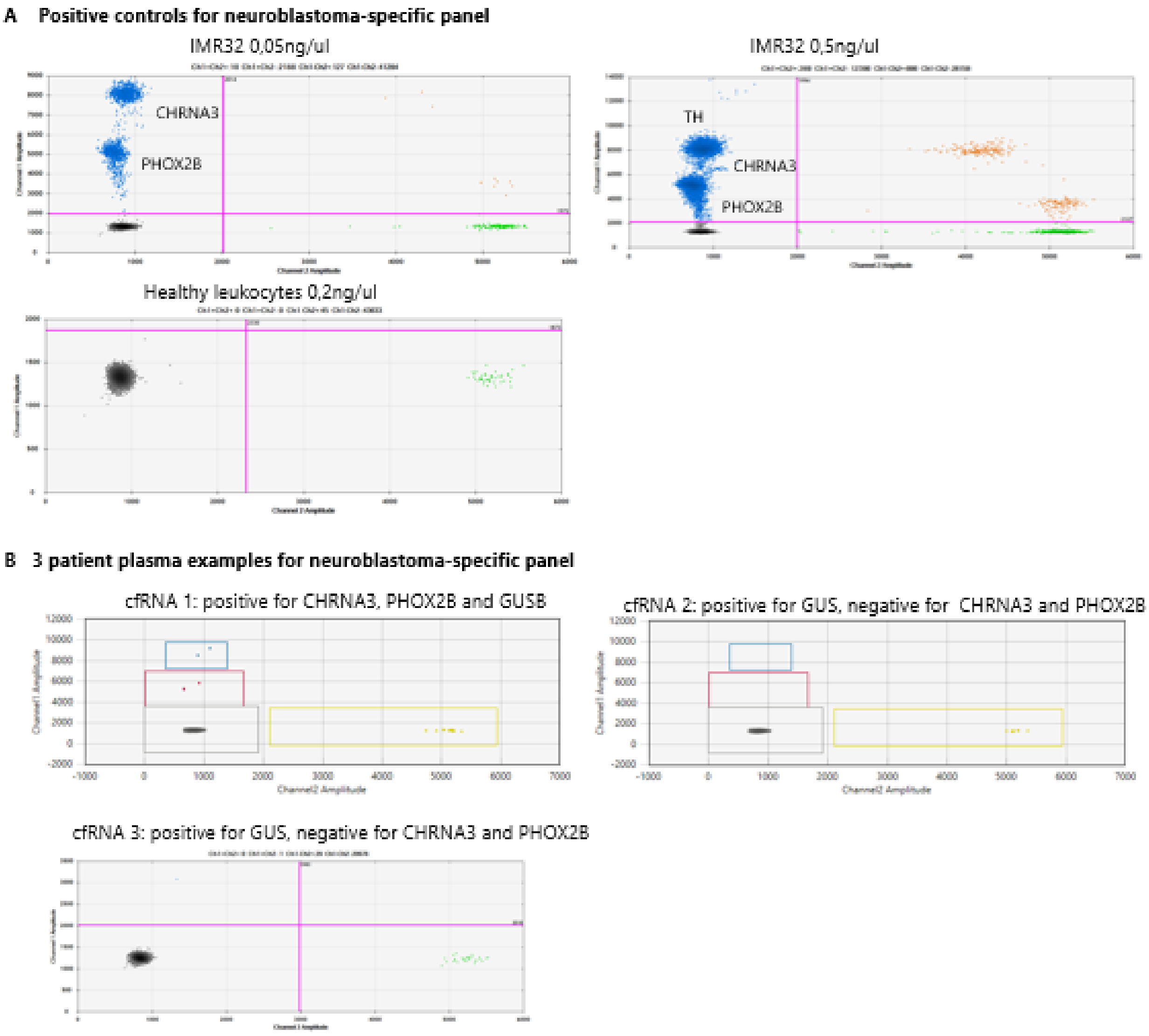

Supplemental Figure S1. 2D plots from the ddPCR assays illustrating gating strategies for the neuroblastoma-specific genes. **A.** Neuroblastoma-specific assay in controls (positive control: neuroblastoma cell line IMR32 0,05 ng/ul, 0,5 ng/ul and negative control: healthy leukocytes 0,2 ng/ul). **B.** cfRNA detection by ddPCR from plasma of 3 patients, cfRNA1 was positive for *CHRNA3*, *PHOX2B* and *GUSB*, cfRNA2 and cfRNA3 only for *GUSB*.

Please note that cfRNA 1 and 2 were analyzed with QX Manager 1.2 Standard Edition software (Bio Rad): pink droplets represent *PHOX2B*, blue droplets *CHRNA3* and yellow droplets *GUSB*. cfRNA 3 was analyzed in Quantasoft 1.7.4 software (Bio Rad), green droplets represent *GUSB*.

Droplets are only counted as positive for *PHOX2B* if they are located exactly above the negative cluster around an amplitude of 5000.

Supplemental Table S2. Results of the neuroblastoma-specific panel and *GUSB* in plasma of 40 healthy controls.

Control ID	GUS copies/ml	PHOX2B copies/ml	TH copies/ml	CHRNA3 copies/ml
**1**	56.49	0.00	0.00	0.00
**2**	196.11	0.00	0.00	0.00
**3**	153.71	0.00	0.00	0.00
**4**	42.40	0.00	0.00	0.00
**5**	97.68	0.00	0.00	0.00
**6**	237.75	0.00	0.00	0.00
**7**	40.66	0.00	0.00	0.00
**8**	111.30	0.00	0.00	0.00
**9**	102.98	0.00	0.00	0.00
**10**	52.47	0.00	0.00	0.00
**11**	104.49	0.00	0.00	0.00
**12**	96.92	0.00	0.00	0.00
**13**	91.62	0.00	0.00	0.00
**14**	58.38	0.00	0.00	0.00
**15**	51.56	0.00	0.00	0.00
**16**	81.77	0.00	0.00	0.00
**17**	36.42	0.00	0.00	0.00
**18**	180.21	0.00	0.00	0.00
**19**	67.39	0.00	0.00	0.00
**20**	60.65	0.00	0.00	0.00
**21**	50.73	0.00	0.00	0.00
**22**	10.60	0.00	0.00	0.00
**23**	35.59	0.00	0.00	0.00
**24**	6.81	0.00	0.00	0.00
**25**	62.09	0.00	0.00	0.00
**26**	12.87	0.00	0.00	0.00
**27**	15.14	0.00	0.00	0.00
**28**	49.97	0.00	0.00	0.00
**29**	24.99	0.00	0.00	0.00
**30**	31.80	0.00	0.00	0.00
**31**	15.14	0.00	0.00	0.00
**32**	15.14	0.00	0.00	0.00
**33**	6.81	0.00	0.00	0.00
**34**	6.81	0.00	0.00	0.00
**35**	29.53	0.00	0.00	0.00
**36**	15.14	0.00	0.00	0.00
**37**	19.69	0.00	0.00	0.00
**38**	10.60	0.00	0.00	0.00
**39**	67.39	0.00	0.00	0.00
**40**	6.06	0.00	0.00	0.00

Supplemental Table S3. Patient characteristics of the first and second cohort.

	NBLnr	Gender	Age at Dx (months)	Stage	Risk	MYCN	LOH1p	Gain17q	ALK	Tumor location	BM	Event	DOD
First cohort	834	0	21	3	2	1	1	1	1	0	1	2	0
	865	0	19	3	2	1	NA	NA	NA	NA	NA	0	0
	2011	1	43	3	2	0	0	NA	1	0	1	0	0
	2012	1	56	3	2	1	1	1	1	0	1	2	1
	2016	1	86	3	2	0	0	1	0	0	1	1	1
	2022	0	26	3	2	1	1	2	1	0	1	2	1
	2024	0	224	3	2	2	0	1	2	0	1	2	1
	2026	1	28	2	2	1	1	2	0	2	0	0	0
	2029	1	78	3	2	2	1	2	2	0	1	2	1
	2032	1	133	3	2	2	0	2	2	0	NA	0	0
	2033	1	5	3	1	0	3	2	1	0	1	0	0
	2034	1	59	3	2	2	0	1	0	0	0	0	0
	2043	0	76	3	2	0	0	1	2	3	1	2	1
	2046	1	79	3	2	0	0	2	0	NA	1	2	1
	2047	0	14	3	2	1	1	1	0	0	1	2	0
	2048	1	50	3	2	0	0	1	0	2	1	4	1
	2049	0	128	3	2	2	0	1	2	1	1	1	1
	2050	0	49	3	2	0	1	1	0	0	1	1	1
	2051	0	57	3	2	0	0	2	0	0	1	2	1
	2052	0	17	3	2	0	0	1	0	1	0	0	0
	2054	0	8	3	2	1	1	1	0	5	0	0	0
	2055	0	35	2	1	2	0	0	2	0	0	1	0
Second cohort	2079	0	28.3	0	0	0	1	1	0	0	0	0	0
	2097	0	5.9	0	0	0	0	1	0	0	0	0	0
	2102	1	20.6	3	2	0	0	0	0	0	0	1	1
	2115	1	3.7	0	0	0	2	1	0	0	0	0	0
	2117	0	13.6	3	2	0	1	1	0	0	1	0	0
	2124	1	32.8	0	0	0	0	0	0	0	0	0	0
	2126	0	13.6	0	0	0	0	0	0	1	0	0	0
	2141	1	45.1	3	2	0	0	1	0	1	1	1	0
	2143	1	56.8	3	2	2	1	1	1	1	1	1	0
	2144	0	52.6	3	2	2	1	1	0	1	1	0	0
	2146	1	32.9	3	2	0	0	1	0	1	1	2	1
	2147	1	62.9	2	0	0	1	1	0	1	0	2	0
	2148	0	17.5	0	0	2	0	1	0	2	0	0	0
	2149	1	10.4	0	0	0	0	1	0	2	0	0	0
	2150	1	95.5	3	2	0	1	1	0	1	1	0	0
	2151	1	36.7	3	2	1	1	1	0	1	1	2	0
	2152	0	12.3	3	2	1	1	1	0	2	0	1	1
	2153	0	33.4	3	2	1	1	1	0	1	0	1	1
	2155	1	1.2	0	0	0	0	1	0	1	0	3	0
	2157	1	9.7	3	2	1	1	1	1	1	1	1	0
	2160	1	8.2	3	2	1	1	1	0	1	0	1	1
	2161	1	11.1	3	1	0	0	1	0	1	1	1	0
	2163	1	52.5	3	2	0	1	1	0	2	0	0	0
	2164	0	29.2	3	2	0	0	1	0	2	0	0	0
	2165	1	141.4	0	0	0	0	0	1	1	0	0	0
	2166	1	13.2	3	2	1	1	1	0	2	1	2	1
	2169	0	0.1	0	0	0	0	0	0	2	0	0	0
	2171	1	9.8	3	1	0	0	1	0	3	1	0	0
	2172	1	153.7	3	2	0	0	0	0	1	1	1	0
	2174	1	1.5	3	1	0	NA	NA	NA	1	1	0	0
	2175	0	138.4	3	2	1	1	0	0	0	1	0	0
	2177	0	30.3	3	2	0	0	1	0	1	1	2	0
	2179	1	11	3	1	0	0	1	0	1	1	1	1
	2181	1	49.3	3	2	0	0	1	0	0	0	0	0
	2183	0	56.8	3	2	0	0	1	0	1	1	0	0
	2184	1	25.1	3	2	0	0	1	0	0	1	1	1
	2187	0	76.3	3	2	0	0	1	0	0	1	1	0
	2193	0	35.8	3	2	1	1	1	1	0	1	1	0
	2194	0	13.33	3	2	1	1	1	0	0	0	1	1
	2196	0	4.4	3	1	0	1	1	0	0	1	1	1
	2211	1	41.73	3	2	0	0	0	1	0	1	1	0

ALK; 0=ALK gene wild type, 1=ALK mutation, 2=gain of ALK
BM; 0=no bone marrow invasion, 1=bone marrow invasion
DOD; 0=did not die of disease, 1=died of disease
Dx; diagnosis
Event; 0=no event, 1=progressive disease, 2=relapse, 3=second malignancy, 4=death to other cause
Gain 17q; 0=no gain of chromosome 17q, 1= gain of 17q, 2=partial gain of 17q
Gender; 0=male, 1=female
LOH1p; 0=no loss of heterozygosity of chromosome 1p, 1=LOH1p, 2=partial LOH1p

MYCN; 0=no aberration in MYCN gene, 1=MYCN amplification, 2=gain in MYCN
NBLnr; unique patient identifier
Stage; number corresponds to International Neuroblastoma Staging System Committee (INSS) stage for neuroblastoma
Tumor location; 0=adrenal, 1=abdominal, 2=thoracic, 3=thoracic-abdominal, 4=paravertebral, 5=adrenal paravertebral thoracic

Supplemental Table S4. Results of ddPCR (copies/ml) of the genes in the first cohort.

NBL ID	Moment	ddPCR (copies/ml plasma)			
		PHOX2B	CHRNA3	TH	GUSBnbl
834	Relapse	0.00	0.00	0.00	2.04
834	After 2nd N8 Relapse therapy	0.00	0.00	0.00	4.18
865	Before ASCT	0.00	0.00	0.00	2.18
2011	3 month after eot	0.00	0.00	0.00	0.00
2012	After IT during relapse	0.00	0.00	0.00	0.00
2016	Relapse therapy	0.00	0.00	0.00	0.00
2016	after Gemcitabine-MIBG	0.00	0.00	0.00	2.17
2022	Relapse	0.00	0.00	0.00	2.21
2022	2 months relapse therapy	0.00	0.00	0.00	2.22
2024	Before start high-dose chemotherapy	0.00	0.00	0.00	0.00
2024	After ASCT	0.00	0.00	0.00	4.53
2026	EOT	0.00	0.00	0.00	13.33
2029	During anti GD2	0.00	0.00	0.00	0.00
2032	After salvage N8	0.00	0.00	0.00	2.22
2033	Before N7	0.00	0.00	0.00	0.00
2034	After ASCT	0.00	0.00	0.00	0.00
2043	Relapse	10.98	0.00	0.00	127.21
2043	After N5/N6 and 2xN8	0.00	0.00	0.00	16.73
2043	After 3rd N5/N6 relapse therapy	0.00	0.00	0.00	95.40
2043	After 2nd relapse Ct	0.00	0.00	0.00	2.23
2046	Diagnosis	0.00	0.00	0.00	8.63
2046	After 2nd N5/N6	0.00	0.00	0.00	37.40
2046	After 2nd N5/N6	0.00	0.00	0.00	2.16
2047	After 3rd N5/N6	0.00	0.00	0.00	8.40
2047	Before ASCT	0.00	0.00	0.00	15.75
2047	After 1st N5/N6	0.00	0.00	0.00	8.48
2048	Diagnosis	0.00	0.00	0.00	4.12
2048	After 1st N5/N6	0.00	0.00	0.00	0.00
2048	After 2nd N5/N6	0.00	0.00	0.00	2.20
2049	Diagnosis	0.00	0.00	0.00	2.23
2049	After 2nd N5/N6	0.00	0.00	0.00	4.22
2050	Diagnosis	2.07	0.00	0.00	2.07
2050	After 1st N5/N6	0.00	0.00	0.00	0.00
2051	Relapse	0.00	0.00	0.00	2.33
2052	Diagnosis	0.00	0.00	0.00	0.00
2052	After 2nd N5/N6	0.00	0.00	0.00	0.00
2054	Diagnosis	0.00	0.00	0.00	0.00
2055	Tumor growth during wait-and-see	0.00	0.00	0.00	13.63

ASCT; autologous stem cell transplantation
EOT; end of treatment
GUSBnbl; GUSB as included in the neuroblastoma-specific assay
GUSBc; GUSB as included in the 'cell cycle' assay
NBL ID; unique patient identifier

Supplemental Table S5. Results of the ddPCR for DNA-based targets (methylated RASSF1A and ACTB), mRNA based targets (neuroblastoma-specific panel with PHOX2B, CHRNA3, TH and GUSB and cell cycle markers CDC6, ATAD2, DHFR, E2F1, H2AFZ, MCM2, GUSB, HPA1A/B and B2M) in the second cohort. Please not that the cell cycle markers are not corrected for platelets in this table.

		DNA targets		Neuroblastoma specific mRNA markers				Cell cycle mRNA markers								
NBL ID	Stage	RASSF1A-M	ACTB /ml plas	PHOX2B	CHRNA3	TH	GUSnbl	CDC6	ATAD2	DHFR	E2F1	H2AFZ	MCM2	GUSB	HPA1AB	B2M
		copies/ml	copies/ml	copies/ml	copies/ml	copies/ml	copies/ml	copies/ml	copies/ml	copies/ml	copies/ml	copies/ml	copies/ml	copies/ml	copies/ml	copies/ml
**2079**	1	3142.9	3661.4	0.0	0.0	0.0	NA	4.6	2.3	29.5	6.8	277.9	13.6	15.8	78.0	3831.3
**2097**	1	17600.0	17285.7	0.0	0.0	0.0	22.1	11.8	17.8	147.6	188.5	1052.5	106.8	78.7	3642.0	73218.7
**2115**	1	785.7	801.4	0.0	0.0	0.0	0.5	0.0	0.0	23.5	2.4	90.9	9.8	4.7	58.8	1438.6
**2124**	1	0.0	3465.0	0.0	0.0	0.0	6.3	0.0	2.3	59.8	11.5	389.2	15.7	18.4	607.3	12720.5
**2126**	1	2734.3	3441.4	0.0	0.0	0.0	NA	0.0	0.0	20.1	4.5	109.8	0.0	6.7	109.8	2877.3
**2148**	1	286.0	9328.0	0.0	0.0	0.0	20.9	2.3	22.3	173.4	89.3	1113.0	44.5	95.4	3331.6	49594.9
**2149**	1	0.0	19305.0	0.0	0.0	0.0	3.5	6.8	12.6	136.3	45.4	483.8	75.1	22.7	376.3	15522.1
**2155**	1	79.8	17847.5	0.0	0.0	0.0	1.4	11.1	6.7	220.3	51.6	885.9	81.8	74.1	645.9	25592.5
**2165**	1	8.3	2282.5	0.0	0.0	0.0	2.6	0.0	0.0	34.1	2.3	102.2	9.1	2.3	236.2	4217.5
**2169**	1	535.3	10413.3	0.0	0.0	0.0	10.4	11.9	9.0	195.4	123.4	1022.2	133.3	62.8	592.9	20216.5
**2102**	4	64606.7	119533.3	0.0	0.0	0.0	14.0	4.9	7.1	1007.0	142.3	908.6	141.6	52.2	832.9	28848.3
**2117**	4	48714.3	55471.4	0.0	0.0	0.0	3.7	0.0	4.6	268.8	34.4	424.8	31.7	16.1	224.1	8783.2
**2141**	4	23430.0	86460.0	0.0	0.0	0.0	27.0	19.4	6.6	855.6	62.1	848.0	79.5	17.7	1953.5	39297.3
**2143**	4	9845.0	22825.0	0.0	0.0	0.0	60.1	9.2	2.2	170.4	305.9	454.3	9.2	77.2	6617.7	124100.8
**2144**	4	8690.0	34782.0	4.4	0.0	0.0	35.1	15.6	8.1	189.3	44.7	493.7	24.5	26.4	446.7	17869.3
**2146**	4	22110.0	69410.0	10.8	2.2	0.0	73.7	58.8	53.1	1181.2	335.4	2210.9	184.0	55.4	3051.4	57393.8
**2150**	4	6160.0	28985.0	0.0	0.0	0.0	19.3	0.0	4.4	63.8	35.2	353.6	26.7	24.2	1415.9	52245.0
**2151**	4	72160.0	108020.0	2.1	0.0	0.0	25.1	6.5	11.0	489.9	171.9	477.8	8.7	35.2	742.0	11039.6
**2152**	4	3476.0	5984.0	0.0	0.0	0.0	22.6	2.1	0.0	61.3	19.0	355.9	12.7	23.2	605.7	28545.5
**2153**	4	52085.0	78705.0	0.0	0.0	0.0	4.8	9.9	2.3	495.2	138.6	380.9	24.8	31.7	2460.8	34678.6
**2157**	4	8052.0	15642.0	0.0	0.0	0.0	21.5	16.3	0.0	107.5	134.8	636.0	34.7	21.0	1468.9	46263.3
**2160**	4	4818.0	22462.0	0.0	0.0	0.0	25.4	8.9	8.7	135.5	87.1	622.4	62.5	24.0	885.9	40205.9
**2161**	4	17343.3	47813.3	4.5	0.0	0.0	56.2	27.7	19.6	246.1	117.4	1113.0	104.5	26.1	742.0	13757.9
**2163**	4	250.8	5456.0	0.0	0.0	0.0	44.4	2.1	3.9	109.8	7.8	583.8	42.3	35.2	170.4	32709.9
**2164**	4	46676.7	103400.0	0.0	0.0	0.0	44.5	2.1	8.5	480.0	230.2	647.4	70.3	36.2	2688.0	126599.5
**2166**	4	591360.0	632720.0	15.5	6.6	0.0	53.2	45.1	44.0	2468.4	160.5	1574.9	118.9	46.0	2067.1	20746.6
**2171**	4	23100.0	102080.0	0.0	2.1	0.0	55.7	19.5	25.5	461.1	238.5	1673.4	103.7	66.0	1953.5	30968.4
**2172**	4	37693.3	84993.3	2.1	0.0	0.0	21.0	8.4	4.4	162.0	53.3	535.3	52.5	31.1	416.4	8359.2
**2174**	4	4253.3	149820.0	46.7	10.6	0.0	81.0	38.4	45.0	1188.8	246.8	1832.4	177.2	98.4	1703.6	10812.4
**2175**	4	17160.0	88366.7	0.0	0.0	0.0	77.2	4.5	6.1	628.5	360.4	583.8	77.2	105.2	3891.9	45809.0
**2177**	4	485100.0	1129700.0	3.3	0.0	0.0	4.7	21.4	20.2	10827.6	192.3	1309.9	128.0	10.1	1294.8	15749.2
**2179**	4	5060.0	30250.0	1.0	0.0	0.0	19.5	13.0	7.2	243.8	198.4	1279.6	62.9	88.6	8480.3	87756.5
**2181**	4	11220.0	30800.0	0.0	0.0	0.0	6.3	13.7	0.0	346.8	295.3	870.8	41.0	39.8	2423.0	53607.9
**2183**	4	22073.3	37033.3	0.4	0.0	0.0	6.1	5.2	11.3	165.1	103.7	581.5	82.5	29.3	1120.6	39297.3
**2184**	4	47.7	4546.7	0.0	0.0	0.0	3.9	8.6	19.6	152.2	12.3	490.6	42.7	12.3	53.8	1718.8
**2187**	4	96525.0	355025.0	0.0	0.0	0.0	184.0	79.5	66.9	863.2	477.0	3740.4	386.9	141.6	15166.2	295297.9
**2193**	4	80685.0	93885.0	2.3	0.0	0.0	46.9	20.2	17.2	112.1	107.5	654.2	38.5	34.5	870.8	12357.1
**2194**	4	6966.7	64460.0	0.0	0.0	0.0	50.1	183.2	96.2	1067.6	487.6	10221.8	1007.0	220.3	4709.6	28545.5
**2196**	4	346.5	49060.0	26.3	0.0	0.0	115.1	35.3	39.4	514.1	137.0	2059.5	165.8	96.9	817.7	34981.4
**2211**	4	7315.0	9680.0	0.4	0.0	0.0	8.0	11.4	0.0	133.3	30.9	817.7	86.3	54.6	2195.8	56333.7

NBL ID; unique patient identifier
Stage; number corresponds to International Neuroblastoma Staging System Committee (INSS) stage for neuroblastoma

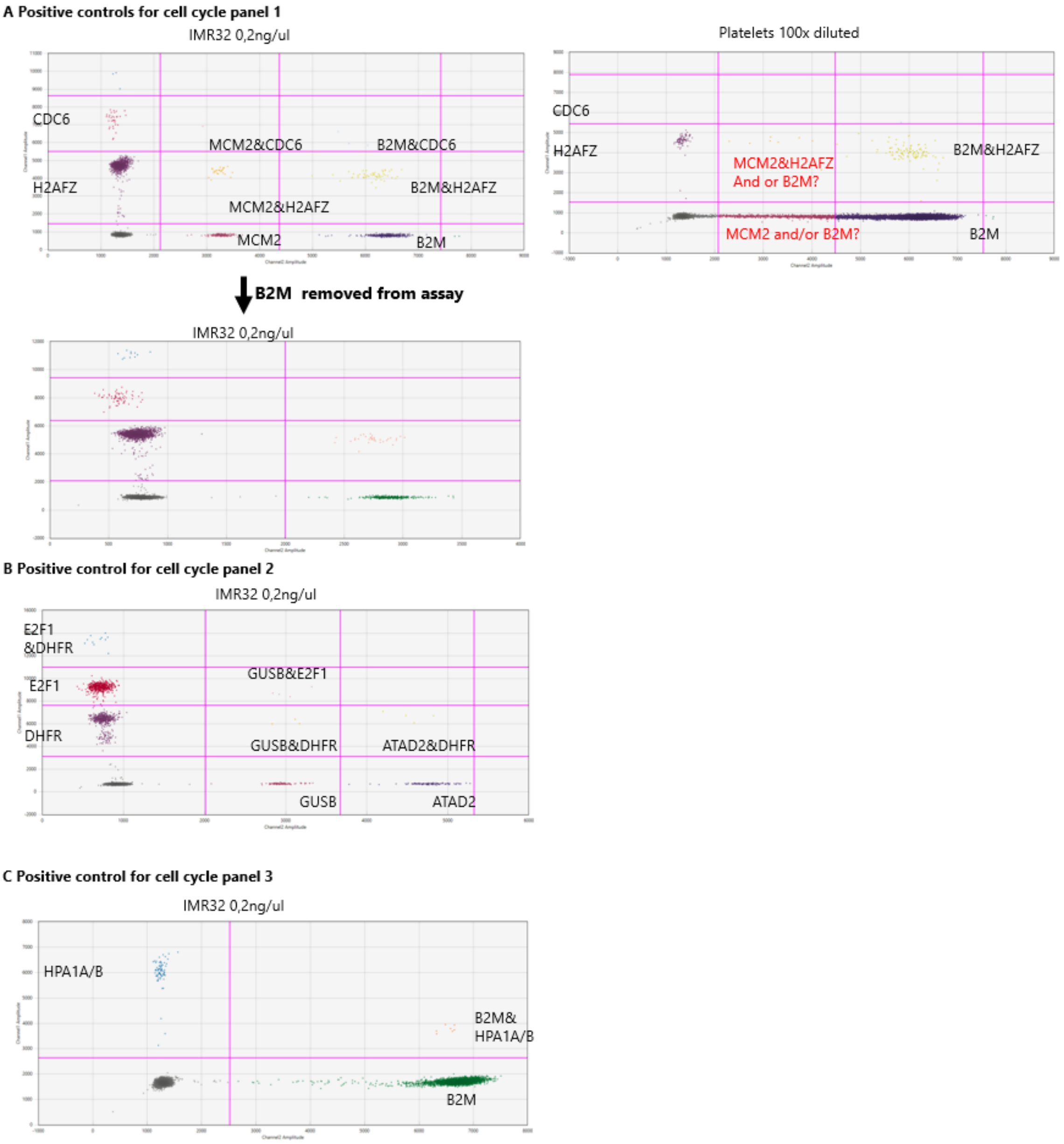

Supplemental Figure S2. 2D plots from the ddPCR assays illustrating gating strategies for the cell cycle genes. **A.** 2D plots for cell cycle panel 1 (top left and right: CDC6 (bright red droplets, H2AFZ dark purple, turquoise double positive for CDC6 and H2AFZ, MCM2 burgundy droplets, B2M dark violet, green double positive for B2M and MCM2, orange positive for MCM2 and H2AFZ, yellow positive for B2M and H2AFZ, pink positive for CDC6 and MCM2, blue positive for B2M and CDC6) (below left (after removal from B2M from this panel): CDC6 bright red droplets, H2AFZ dark purple, turquoise double positive for CDC6 and H2AFZ, MCM2 green droplets, orange positive for MCM2 and H2AFZ. **B.** 2D plot for cell cycle panel 2 (bright red droplets E2F1, DHFR dark purple, turquoise E2F1 and DHFR double positive, GUSB burgundy, ATAD2 dark violet, orange GUSB and DHFR, light pink GUSB and E2F1, yellow ATAD2 and DHFR). **C.** 2D plot for cell cycle panel 3 (turquoise droplets positive for HPA1A/B, dark green B2M, orange B2M and HPA1A/B).
Please note that these 2D plots were all generated in QX Manager 1.2 Standard Edition (BioRad).

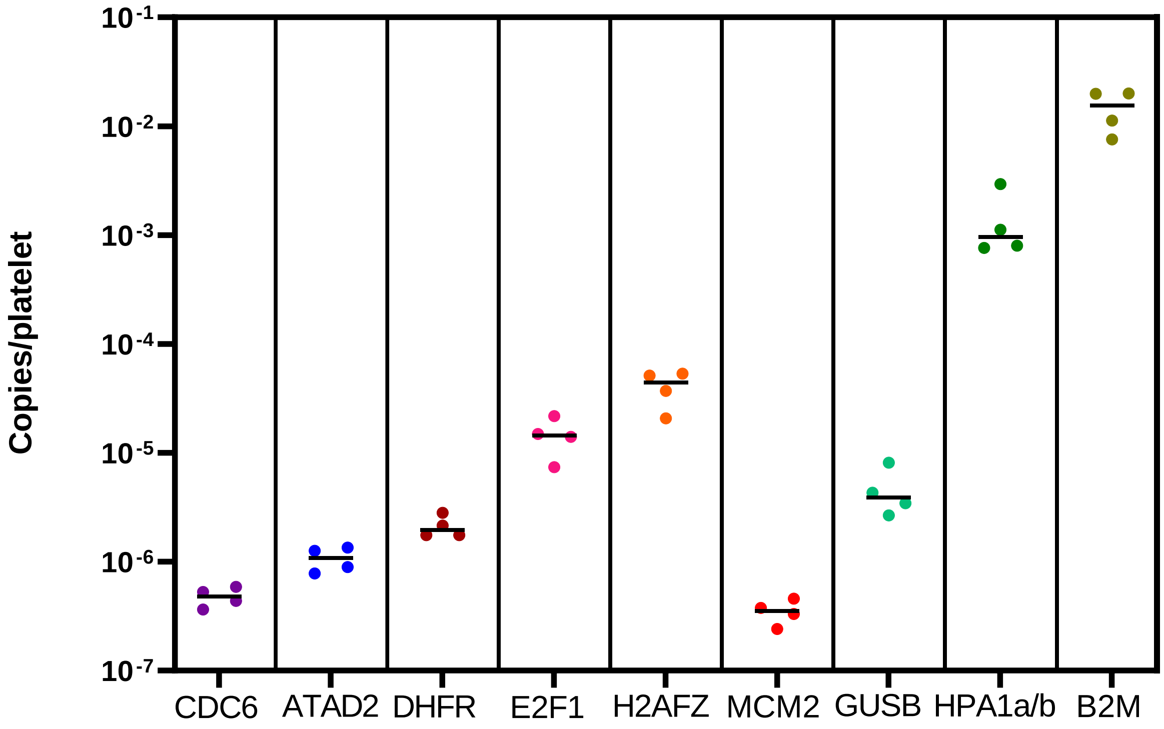

Supplemental Figure S3. Expression of cell cycle genes (CDC6, ATAD2, DHFR, E2F1, H2AFZ and MCM2), reference genes (GUSB and B2M) and platelet-specific gene HPA1a/b in platelets from 4 healthy donors as measured by ddPCR.

Supplemental Table S6. Overview of number of leukocytes and platelets recovered at the different centrifugation steps during the preparation of platelet poor plasma, and their percentages to whole blood.

PLATELET POOR PLASMA											
		Whole blood		After 1x centrifugation in plasma				After 1x centrifugation in cellular fraction			
Healthy control ID		number/ul	Total in 9 ml	number/ul	PPP volume (ml)	Total in PPP	% of whole blood	number/ul	Cell fraction	Total in cell fraction	% of whole blood
**Control 1**	Leukocytes	8 330	74 970 000	0.70	4	2800	0.00	13 050.00	6 000.00	78 300 000.00	104.44
	Platelets	298 000	2 682 000 000	236 000.00	4	944 000 000.00	35.20	351 000.00	6 000.00	2 106 000 000.00	78.52
**Control 2**	Leukocytes	5 590	50 310 000	0.80	3.5	2 800.00	0.01	8 120.00	6 000.00	48 720 000.00	96.84
	Platelets	189 000	1 701 000 000	234 000.00	3.5	819 000 000.00	48.15	210 000.00	6 000.00	1 260 000 000.00	74.07
**Control 3**	Leukocytes	6 840	61 560 000	1.50	3	4 500.00	0.01	8 650.00	7 000.00	60 550 000.00	98.36
	Platelets	240 000	2 160 000 000	159 000.00	3	477 000 000.00	22.08	276 000.00	7 000.00	1 932 000 000.00	89.44
**Control 4**	Leukocytes	3 730	33 570 000	0.90	4	3 600.00	0.01	6 010.00	7 000.00	42 070 000.00	125.32
	Platelets	257 000	2 313 000 000	175 000.00	4	700 000 000.00	30.26	275 000.00	7 000.00	1 925 000 000.00	83.23

Supplemental Table S7. Expression of cell cycle genes in 4 samples of platelets isolated from healthy controls and the corresponding correction co-efficients, as calculated by the following formula:

$$Correction\ co-efficient = mean\ (copies/mL) + (3 * Standard\ deviation)$$

Control ID	CDC6 (copies)	Ratio marker/ HPA1ab	ATAD2 (copies)	Ratio marker/ HPA1ab	DHFR (copies)	Ratio marker/ HPA1ab	E2F1 (copies)	Ratio marker/ HPA1ab	H2AFZ (copies)	Ratio marker/ HPA1ab	MCM2 (copies)	Ratio marker/ HPA1ab	GUSB (copies)	Ratio marker/ HPA1ab	B2M (copies)	Ratio marker/ HPA1ab	HPA1a/b (copies)
Tr1	352.84	0.000472	522.45	0.000698	1441.66	0.001927	9964.41	0.013320	24956.46	0.033360	222.61	0.000298	2301.81	0.003077	7526309.56	10.060729	748087.91
Tr3	215.04	0.000174	526.99	0.000426	1038.84	0.000841	8268.34	0.006691	12266.22	0.009926	142.20	0.000115	1574.92	0.001275	8328914.00	6.740196	1235707.97
Tr2	246.84	0.000524	564.85	0.001199	1176.65	0.002498	9101.23	0.019325	21443.17	0.045531	192.32	0.000408	3407.28	0.007235	4452183.12	9.453376	470962.23
Tr4	305.90	0.000571	885.89	0.001653	1228.14	0.002291	5179.07	0.009661	37525.54	0.070000	263.50	0.000492	3028.70	0.005650	14007719.00	26.129944	536079.19
Mean copies	280.15	0.000435	625.05	0.000994	1221.32	0.001889	8128.26	0.012249	24047.85	0.039704	205.16	0.000328	2578.18	0.004309	8578781.42	13.096061	747709.33
Standard deviation	215.04	0.000174	522.45	0.000426	1038.84	0.000841	5179.07	0.006691	12266.22	0.009926	142.20	0.000115	1574.92	0.001275	4452183.12	6.740196	470962.23
Minimum	352.84	0.000571	885.89	0.001653	1441.66	0.002498	9964.41	0.019325	37525.54	0.070000	263.50	0.000492	3407.28	0.007235	14007719.00	26.129944	1235707.97
Maximum	61.36	0.000179	174.93	0.000543	167.22	0.000738	2084.50	0.005441	10457.29	0.025025	51.11	0.000163	811.02	0.002651	3986322.76	8.808358	346178.71
Correction co-efficient		0.000971		0.002624		0.004103		0.028571		0.114779		0.000816		0.012262		39.521135	

Supplemental Table S8. Expression of cell cycles genes in plasma from the 20 healthy controls (as shown previously in Supplemental Table S6) with adjacent the levels after correction for platelets using the correction co-efficient per marker and below the calculated thresholds for positivity. The following 2 formulas were used:

$$'After\ correction'(copies/ml) = marker(copies/mL) - (correction\ coefficient * HPA1a/b(copies/ml))$$

$$'Threshold\ for\ positivity' = mean(copies/mL) + (3 * Standard\ deviation)$$

	CDC6 (copies/ml)	After subtraction platelets	ATAD2 (copies/ml)	After subtraction platelets	DHFR (copies/ml)	After subtraction platelets	E2F1 (copies/ml)	After subtraction platelets	H2AFZ (copies/ml)	After subtraction platelets	MCM2 (copies/ml)	After subtraction platelets	GUS (copies/ml)	After subtraction platelets	B2M (copies/ml)	After subtraction platelets	HPA1A/B (copies/ml)
1	15.45	11.29	12.11	0.87	93.13	75.55	127.21	4.76	1082.76	590.86	NA		56.49	3.94	NA		4285.60
2	28.92	-50.41	55.05	-159.33	106.00	-229.21	434.62	-1899.61	1658.21	-7719.13	NA		196.11	-805.69	NA		81699.07
3	23.02	-75.35	36.42	-229.42	224.88	-190.79	286.21	-2608.31	2074.66	-9553.59	NA		153.71	-1088.56	NA		101309.88
4	6.75	2.14	24.53	12.08	87.08	67.60	129.48	-6.16	1173.62	628.71	NA		42.40	-15.81	NA		4747.48
5	10.37	-18.52	18.02	-60.06	127.96	5.87	199.89	-650.29	3377.00	-38.48	81.02	56.74	97.68	-267.20	843643.27	-332384.70	29756.94
6	23.32	-2.78	22.72	-47.82	150.68	40.39	351.33	-416.65	2218.52	-866.70	146.13	124.20	237.75	-91.85	664874.49	-397440.85	26879.68
7	4.94	-3.67	2.91	-20.34	69.74	33.39	203.68	-49.43	686.76	-330.06	54.37	47.14	40.66	-67.97	132429.73	-217685.47	8858.94
8	5.91	-61.95	9.24	-174.15	145.38	-141.37	259.71	-1737.04	1892.94	-6128.64	38.39	-18.64	111.30	-745.65	605436.33	-2156583.56	69887.16
9	3.32	-26.90	2.28	-79.38	89.35	-38.34	166.58	-722.55	999.47	-2572.44	29.91	4.51	102.98	-278.62	201029.70	-1028862.15	31119.85
10	11.51	6.24	4.37	-9.86	83.29	61.05	48.08	-106.81	688.27	66.01	45.96	41.54	52.47	-14.00	137124.21	-77134.32	5421.37
11	5.05	-10.32	5.21	-36.32	151.43	86.51	117.36	-334.77	1590.07	-226.30	121.15	108.23	104.49	-89.56	79200.40	-546219.06	15824.94
12	24.38	-60.68	3.35	-226.53	100.70	-258.74	274.85	-2228.11	938.90	-9116.32	18.93	-52.56	96.92	-977.29	586734.13	-2875516.16	87605.03
13	13.25	-38.14	18.32	-120.56	89.35	-127.81	215.04	-1297.12	1370.48	-4704.36	19.91	-23.27	91.62	-557.37	499431.97	-1592281.90	52926.46
14	6.35	-26.00	23.85	-63.57	109.03	-27.66	353.60	-598.26	726.89	-3097.05	25.44	-1.74	58.38	-350.14	311879.97	-1004792.57	33315.66
15	4.59	-26.66	3.03	-81.41	102.98	-29.06	279.40	-640.01	908.61	-2784.97	43.61	17.35	51.56	-343.03	188309.17	-1083476.80	32179.90
16	11.51	-44.07	13.10	-137.10	134.78	-100.09	91.62	-1543.85	878.32	-5691.90	86.32	39.61	81.77	-620.13	217990.39	-2044292.42	57242.35
17	6.04	0.00	6.83	-9.50	61.48	35.95	25.06	-152.76	984.33	269.95	33.24	28.16	36.42	-39.90	341561.19	95582.82	6223.97
18	24.23	-77.01	37.56	-236.03	198.38	-229.41	404.33	-2574.56	2135.23	-9831.96	86.32	1.24	180.21	-1098.26	789202.46	-3331384.10	104262.86
19	4.55	-5.01	12.27	-13.56	128.72	88.33	137.81	-143.43	730.67	-399.13	68.30	60.26	67.39	-53.31	200348.24	-188668.65	9843.26
20	16.51	7.02	6.06	-19.57	212.01	171.93	69.74	-209.33	1060.04	-61.07	104.49	96.52	60.65	-59.12	269251.07	-116773.37	9767.54
Mean	12.50	-25.04	15.86	-85.58	123.32	-35.30	208.78	-895.72	1358.79	-3078.33	62.72	33.08	96.05	-377.98	379277.92	-1056119.58	38657.90
Standard deviation	8.23	28.43	14.14	81.99	46.04	124.80	119.07	892.71	693.21	3692.53	38.29	49.05	55.97	384.44	248706.29	1058502.97	33957.73
Minimum	3.32	-77.01	2.28	-236.03	61.48	-258.74	25.06	-2608.31	686.76	-9831.96	18.93	-52.56	36.42	-1098.26	79200.40	-3331384.10	4285.60
Maximum	28.92	11.29	55.05	12.08	224.88	171.93	434.62	4.76	3377.00	628.71	146.13	124.20	237.75	3.94	843643.27	95582.82	104262.86
Control threshold	37.19	60.24	58.29	160.40	261.45	339.11	565.99	1782.41	3438.40	7999.25	177.58	180.22	263.95	775.33	1125396.80	2119389.33	140531.09

Supplemental Table S9. Overview of the cell cycle marker results and the levels corrected for presence of platelets using the above-mentioned correction co-efficients. Cells in red are positive according to the thresholds (as shown in Supplemental Table S9).

NBL ID	Stage	CDC6	Corrected for HPA	ATAD2	Corrected for HPA	DHFR	Corrected for HPA	E2F1	Corrected for HPA	H2AFZ	Corrected for HPA	MCM2	Corrected for HPA	GUS	Corrected for HPA	B2M	Corrected for HPA	HPA1AB
**2079**	1	4.550616	4.4748885	2.26395	2.0593073	29.45407	29.1340801	6.791851	4.5636293	277.8829	268.93137	13.62913	13.565493	15.82494	14.86863644	3831.3	749.090115	77.98892
**2097**	1	11.81191	8.2755257	17.79359	8.2369628	147.6489	132.705776	188.5363	84.480546	1052.472	634.44595	106.7615	103.789656	78.7461	34.0878069	73218.73	-70717.504	3642.007
**2115**	1	0	-0.0571263	0	-0.154376	23.54811	23.306722	2.354811	0.6739101	90.86088	84.108154	9.767545	9.71953735	4.709622	3.988219148	1438.631	-886.49311	58.83242
**2124**	1	0	-0.5896432	2.301809	0.7083757	59.81675	57.3251847	11.50904	-5.8407963	389.1874	319.48748	15.6735	15.1779829	18.39933	10.95318519	12720.52	-11278.823	607.2535
**2126**	1	0	-0.1066063	0	-0.28809	20.06511	19.6146417	4.459755	1.3229382	109.7902	97.188617	0	-0.08958883	6.685846	5.33959862	2877.261	-1461.7728	109.7902
**2148**	1	2.347239	-0.8877108	22.26092	13.518887	173.3928	159.723432	89.34653	-5.8396288	1113.046	730.65201	44.52183	41.8032737	95.40392	54.55226661	49594.9	-82072.34	3331.566
**2149**	1	6.829709	6.4643072	12.64481	11.657354	136.2913	134.747298	45.43044	34.67873	483.8342	440.64107	75.11166	74.8045874	22.71522	18.10083961	15522.07	649.654073	376.3155
**2155**	1	11.05474	10.427601	6.731277	5.0365155	220.3376	217.687632	51.63927	33.186132	885.8936	811.76133	81.77479	81.2477626	74.12733	66.20768375	25592.48	66.9918101	645.8694
**2165**	1	0	-0.2293874	0	-0.619889	34.07283	33.1035443	2.271522	-4.4780421	102.2185	75.103296	9.086088	8.89331756	2.271522	-0.62523189	4217.459	-5118.9449	236.2383
**2169**	1	11.88763	11.311958	9.010371	7.454687	195.3509	192.918358	123.4194	106.48055	1022.185	954.13619	133.2626	132.778844	62.84544	55.57570388	20216.55	-3214.2375	592.8672
**2102**	4	4.868629	4.0598913	7.109864	4.9243568	1007.041	1003.62407	142.3487	118.55217	908.6088	813.01036	141.5915	140.911899	52.16929	41.95637425	28848.33	-4068.4799	832.8914
**2117**	4	0	-0.2176239	4.588474	4.0003744	268.7968	267.877191	34.3757	27.972267	424.7746	399.04994	31.72559	31.5427058	16.05209	13.30388639	8783.218	-74.395738	224.1235
**2141**	4	19.38365	17.486797	6.647988	1.5219803	855.6066	847.591373	62.08827	6.2745646	848.0349	623.81308	79.50327	77.9092067	17.71787	-6.23605478	39297.33	-37907.549	1953.509
**2143**	4	9.237523	2.8117354	2.150374	-15.21447	170.3642	143.211724	305.8983	116.82397	454.3044	-305.26868	9.237523	3.83747898	77.23175	-3.91449872	124100.8	-137438.19	6617.701
**2144**	4	15.59778	15.164007	8.101762	6.9295353	189.2935	187.460556	44.67327	31.909667	493.6774	442.40192	24.45672	24.0921863	26.42537	20.94753672	17869.31	213.926869	446.7327
**2146**	4	58.83242	55.8695	53.0779	45.070994	1181.191	1168.6715	335.4281	248.24621	2210.948	1860.7102	183.9933	181.50333	55.42514	18.00873242	57393.79	-63201.43	3051.411
**2150**	4	0	-1.3748538	4.399181	0.683819	63.75405	57.94455	35.20859	-5.2455273	353.6003	191.08291	26.65252	25.4971378	24.15385	6.79189621	52245.01	-3713.5698	1415.915
**2151**	4	6.534412	5.8139	10.97902	9.0319349	489.8916	486.847027	171.8785	150.67794	477.7768	392.60727	8.707501	8.1020041	35.20859	26.10981276	11039.6	-18286.288	742.0305
**2152**	4	2.127659	1.5394862	0	-1.58946	61.25538	58.7700287	19.00507	1.6984927	355.8718	286.34564	12.72052	12.22624	23.24524	15.81766773	28545.46	4605.96213	605.7392
**2153**	4	9.918979	7.5295275	2.26395	-4.19323	495.1918	485.09507	138.5628	68.254882	380.8585	98.40858	24.83531	22.8272818	31.72559	1.551070939	34678.57	-62575.64	2460.816
**2157**	4	16.27924	14.852922	0	-3.85444	107.5187	101.491739	134.777	92.808528	636.0262	467.42527	34.67857	33.4799325	21.04944	3.037570079	46263.33	-11789.95	1468.918
**2160**	4	8.934653	8.0744505	8.707501	6.3829162	135.5341	131.899325	87.07501	61.764145	622.397	520.71505	62.46686	61.7439658	24.00242	13.13958872	40205.94	5194.42406	885.8936
**2161**	4	27.71257	26.992057	19.61081	17.663719	246.0816	243.036999	117.362	96.161416	1113.046	1027.8763	104.49	103.884515	26.1225	17.02372476	13757.85	-15568.033	742.0305
**2163**	4	2.112515	1.9470919	3.907018	3.4599823	109.7902	109.091226	7.798892	2.9314181	583.7812	564.22693	42.32603	42.1870095	35.20859	33.11958579	32709.92	25976.9331	170.3642
**2164**	4	2.067085	-0.5429316	8.480349	1.4271216	480.0483	469.019585	230.1809	153.38297	647.3838	338.86153	70.34146	68.148083	36.19292	3.233057263	126599.5	20367.9719	2687.968
**2166**	4	45.12757	43.120431	43.99181	38.567778	2468.387	2459.90599	160.5209	101.4622	1574.922	1337.664	118.8763	117.189577	46.03618	20.68958268	20746.57	-60946.968	2067.085
**2171**	4	19.53509	17.638232	25.51676	20.390756	461.119	453.103719	238.5098	182.69611	1673.355	1449.1327	103.7328	102.138775	66.02557	42.07164642	30968.42	-46236.463	1953.509
**2172**	4	8.404631	8.0002626	4.43704	3.3442861	162.0352	160.326559	53.30505	41.40678	535.322	487.5228	52.54788	52.2080559	31.11985	26.01339423	8359.201	-8099.2037	416.4457
**2174**	4	38.38872	36.734486	44.97614	40.50578	1188.763	1181.77314	246.8387	198.16398	1832.361	1636.8188	177.1787	175.788545	98.43262	77.54256793	10812.44	-56517.392	1703.642
**2175**	4	4.543044	0.764034	6.064964	-4.147315	628.4544	612.48606	360.4148	249.22008	583.7812	137.07571	77.23175	74.0559785	105.2472	57.5250226	45809.03	-108002.25	3891.874
**2177**	4	21.35231	20.095088	20.21655	16.819076	10827.59	10822.2758	192.3222	155.32939	1309.911	1161.2989	127.9624	126.905876	10.14613	-5.73030798	15749.22	-35421.457	1294.768
**2179**	4	13.02339	4.7889741	7.170438	-15.082	243.81	209.015157	198.3796	-43.912458	1279.624	306.25811	62.92116	56.0011948	88.58936	-15.396679	87756.47	-247396.5	8480.349
**2181**	4	13.70485	11.352158	0	-6.357839	346.7857	336.8443	295.2979	226.07156	870.7501	592.64554	41.03883	39.0616981	39.75164	10.04133872	53607.92	-42150.071	2422.957
**2183**	4	5.156355	4.0682353	11.28189	8.3413922	165.0639	160.466038	103.7328	71.715675	581.5096	452.88627	82.53197	81.6175421	29.30263	15.56162177	39297.33	-4990.7401	1120.618
**2184**	4	8.556066	8.5038659	19.61081	19.469742	152.192	151.971399	12.26622	10.73026	490.6488	484.47831	42.70461	42.660746	12.26622	11.6070216	1718.785	-405.84544	53.75935
**2187**	4	79.50327	64.776894	66.93418	27.138085	863.1784	800.951461	477.0196	43.706256	3740.44	1999.6788	386.9159	374.540299	141.5915	-44.3763478	295297.9	-304087.31	15166.2
**2193**	4	20.21655	19.371047	17.18785	14.903002	112.0618	108.489064	107.5187	82.640507	654.1983	554.25451	38.46444	37.7539071	34.45142	23.77427927	12357.08	-22055.948	870.7501
**2194**	4	183.2361	178.66306	96.1611	83.803049	1067.615	1048.29176	487.6201	353.06144	10221.85	9681.2833	1007.041	1003.19837	220.3376	162.5882456	28545.46	-157584.13	4709.622
**2196**	4	35.28431	34.490275	39.37305	37.227277	514.1211	510.765926	137.0485	113.68462	2059.513	1965.653	165.8211	165.153824	96.91827	86.891047	34981.44	2663.11695	817.7479
**2211**	4	11.35761	9.2254837	0	-5.761791	133.2626	124.253238	30.8927	-31.843634	817.7479	565.71566	86.31784	84.5260594	54.59225	27.66728939	56333.75	-30446.933	2195.805

NBL ID; unique patient identifier

Stage; number corresponds to International Neuroblastoma Staging System Committee (INSS) stage for neuroblastoma

Supplemental Table S10. Levels of the neuroblastoma-specific markers and cell cycle genes in sequential plasma samples from 11 patients with metastatic neuroblastoma.

NBL ID	Time point	CDC6 (copies/ml)	ATAD2 (copies/ml)	DHFR (copies/ml)	E2F1 (copies/ml)	H2AFZ (copies/ml)	MCM2 (copies/ml)	GUS (copies/ml)	HPA1AB (copies/ml)	B2M (copies/ml)
2141	Diagnosis	19.38365	6.647988	855.6066	62.08827	848.0349	79.50327	17.71787	1953.509	39297.33
	After 2CT 1L	40.81168	74.80879	475.5053	57.69666	1249.337	129.4768	47.02051	209.7372	4255.318
	After 4Ct 1L	0	0	8.556066	2.142802	43.91609	2.309381	6.428407	4.149314	585.2955
	After 6Ct 1L	2.233663	6.57227	15.29491	2.188233	62.46686	4.459755	6.57227	8.783218	750.3594
	1Ct 2L	0	0	25.81963	0	60.87679	2.248807	4.300748	51.94214	3323.994
	1IT	4.626333	6.04982	111.3046	8.101762	212.7659	64.81409	6.04982	75.7174	5572.801
	2IT	4.543044	4.149314	16.58211	0	49.97348	4.543044	4.149314	39.1459	2067.085
	3IT	2.097372	2.082229	35.36003	0	60.95251	0	0	29.37835	2460.816
	End IT	0	4.702051	49.36774	0	84.04631	2.142802	2.347239	8.404631	3823.729
2144	Diagnosis	27.71257	19.61081	246.0816	117.362	1113.046	104.49	26.1225	742.0305	13757.85
	After 4Ct 1L	18.09646	33.39137	287.7261	57.84809	916.1805	119.6335	40.0545	100.7041	7798.892
	After 6Ct 1L	0	2.157946	88.58936	0	94.64675	6.458694	6.473838	12.79624	5451.653
	1IT	2.180661	6.640416	28.77261	4.429468	74.05162	8.707501	2.210948	6.564699	1559.778
	2IT	0	2.074657	14.53774	0	61.17966	4.702051	0	11.13046	2112.515
	End IT	0	2.347239	18.77792	0	57.54522	0	0	0	1514.348
2161	Diagnosis	27.71257	19.61081	246.0816	117.362	1113.046	104.49	26.1225	742.0305	13757.85
	After 2CT 1L	19.23222	55.12227	239.267	50.57922	602.7105	57.77238	45.96046	82.53197	7155.294
	After 4Ct 1L	2.210948	17.33928	77.98892	4.331035	281.6687	4.421896	17.33928	14.84061	2377.526
	After 6Ct 1L	197.6224	174.15	1340.198	221.0948	7276.442	741.2733	264.2537	145.3774	6890.283
	1Ct 2L	6.087679	0	39.6002	8.328914	324.8276	34.45142	20.82229	195.9188	5035.207
	End 2L	4.081168	8.556066	183.9933	2.135231	81.77479	10.22185	2.135231	32.93707	3157.416
	Relapse	0	2.188233	183.9933	0	202.9226	2.029226	10.90331	20.898	8858.936
2175	Diagnosis	4.543044	6.064964	628.4544	360.4148	583.7812	77.23175	105.2472	3891.874	45809.03
	After 2CT 1L	50.88209	54.06222	610.2822	108.2759	1537.063	133.2626	54.06222	598.1675	15597.78
	After 4Ct 1L	0	2.036798	16.27924	2.036798	45.12757	0	6.102822	0	802.6044
	After 6Ct 1L	0	0	9.010371	22.48807	36.42007	0	0	58.22668	779.8892
	After MAT	2.173089	2.097372	255.9248	14.68918	50.0492	32.6342	0	21.12515	1007.041
	1IT	0	0	181.7218	6.117966	163.5496	52.54788	4.081168	55.95516	2869.689
	3IT	0	0	238.5098	10.82759	112.0618	28.54546	4.323464	101.4613	4346.179
	End IT	6.405692	3.96002	31.72559	57.46951	271.8255	29.90837	17.79359	739.0018	16809.26
2179	Diagnosis	13.02339	7.170438	243.81	198.3796	1279.624	62.92116	88.58936	8480.349	87756.47
	After 2CT 1L	33.76996	64.35979	1014.613	84.04631	1097.902	130.9911	68.82712	817.7479	6647.988
	After 3Ct 1L	232.4524	193.8365	2430.529	370.2581	9540.392	954.0392	466.4192	2347.239	34375.7
	After 4Ct 1L	14.99205	2.082229	154.4635	45.88474	662.5273	111.3046	14.61346	454.3044	3581.433
	After 5Ct 1L	0	0	35.13287	2.195805	13.55341	4.512757	0	0	177.1787
2181	Diagnosis	13.70485	0	346.7857	295.2979	870.7501	41.03883	39.75164	2422.957	53607.92
	After 2CT 1L	74.88451	62.76972	736.7303	290.7548	4141.742	402.0594	121.1478	233.2096	9464.675
	After 6Ct 1L	152.192	151.4348	885.8936	212.7659	8556.066	555.7657	225.6379	498.9777	10373.28
	1IT	2.104944	2.074657	258.9535	14.53774	204.437	10.52472	8.253197	281.6687	5421.366
	End IT	0	9.086088	81.77479	6.829709	180.9646	18.92935	9.086088	159.0065	9313.24
2183	Diagnosis	5.156355	11.28189	165.0639	103.7328	581.5096	82.53197	29.30263	1120.618	39297.33
	After 2CT 1L	6.170968	0	78.7461	30.58983	436.8894	30.8927	30.58983	128.7196	3649.579
	After 4Ct 1L	16.27924	26.80396	241.5385	91.61805	1044.9	152.9491	44.74898	121.905	4096.311
	After 6Ct 1L	0	0	17.18785	6.451122	147.6489	16.65783	8.631784	60.0439	1219.05
	1Ct 2L	0	0	24.91102	14.53774	56.71233	4.361322	4.149314	47.32338	1256.909
	After MAT	0	0	216.5518	0	299.0837	77.98892	9.161805	65.57127	3339.137
	1IT	2.256379	4.353751	235.4811	2.180661	168.8498	18.02074	15.2192	44.52183	3702.581
	End IT	6.201255	6.943286	60.19533	9.237523	194.5937	10.29757	2.316952	45.88474	6814.566
2187	Diagnosis	79.50327	66.93418	863.1784	477.0196	3740.44	386.9159	141.5915	15166.2	295297.9
	2Ct 1L	17.11213	8.328914	257.4392	47.70196	1143.333	96.1611	26.95539	105.2472	3263.42
	6Ct 1L	24.53244	14.53774	146.8918	33.16422	728.4014	49.14059	41.41742	220.3376	3005.981
	End of 2L	4.512757	4.103883	135.5341	2.051942	87.83218	13.55341	6.163396	4.111455	1332.626
	End of 3L	0	0	243.81	6.587414	158.2494	15.14348	4.391609	57.77238	3929.733
2193	Diagnosis	20.21655	17.18785	112.0618	107.5187	654.1983	38.46444	34.45142	870.7501	12357.08
	After 2CT 1L	38.61587	33.9214	660.2557	106.0044	1219.05	148.4061	80.26044	890.6259	14102.37
	After 4Ct 1L	19.08078	8.328914	529.2646	52.01785	1067.615	243.81	49.89777	171.8785	3763.155
	After 6Ct 1L	13.32626	23.69955	224.8807	47.47481	497.4633	71.17436	47.47481	458.8474	6246.686
	End of 2L	23.69955	10.67615	166.5783	38.38872	1166.048	77.98892	57.54522	137.0485	6155.825
	After MAT	0	6.708562	105.2472	26.80396	87.07501	4.058453	2.233663	673.1277	17112.13
	End of 3L	2.226092	2.142802	203.6798	2.142802	71.25007	2.226092	4.285605	22.6395	4785.34
2194	Diagnosis	183.2361	96.1611	1067.615	487.6201	10221.85	1007.041	220.3376	4709.622	28545.46
	After 2CT 1L	749.6023	893.4653	11584.76	779.8892	26425.37	3119.557	1317.483	2657.681	54819.4
	After 4Ct 1L	62.76972	65.57127	1650.639	167.3355	3498.144	514.1211	152.192	727.6442	11509.04
	After 6Ct 1L	258.9535	157.4922	1650.639	351.3287	10373.28	1173.62	284.6974	586.8099	9237.523
2196	Diagnosis	35.28431	39.37305	514.1211	137.0485	2059.513	165.8211	96.91827	817.7479	34981.44
	After 2CT 1L	8.328914	14.76489	5898.385	19.00507	1696.07	545.9225	101.4613	178.6931	14916.33

1 IT= at first course of anti-GD2 immunotherapy
After 2Ct 1L= after 2 courrses of chemotherapy in first line therapy
After MAT= after myeoloablative therapy and autologous stem cell treatment
End of 2L= end of second line therapy
End of 3L= end of third line therapy
End of IT=at the end of anti-GD2 immunotherapy courses

A No event

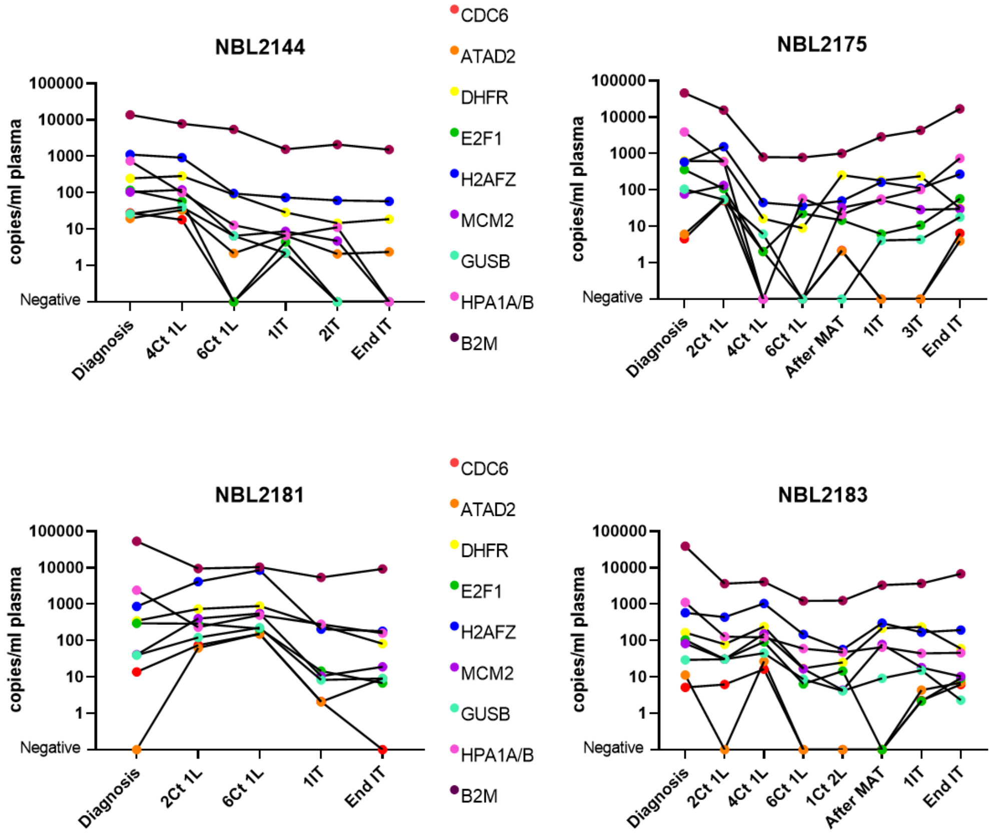

B Event but salvaged

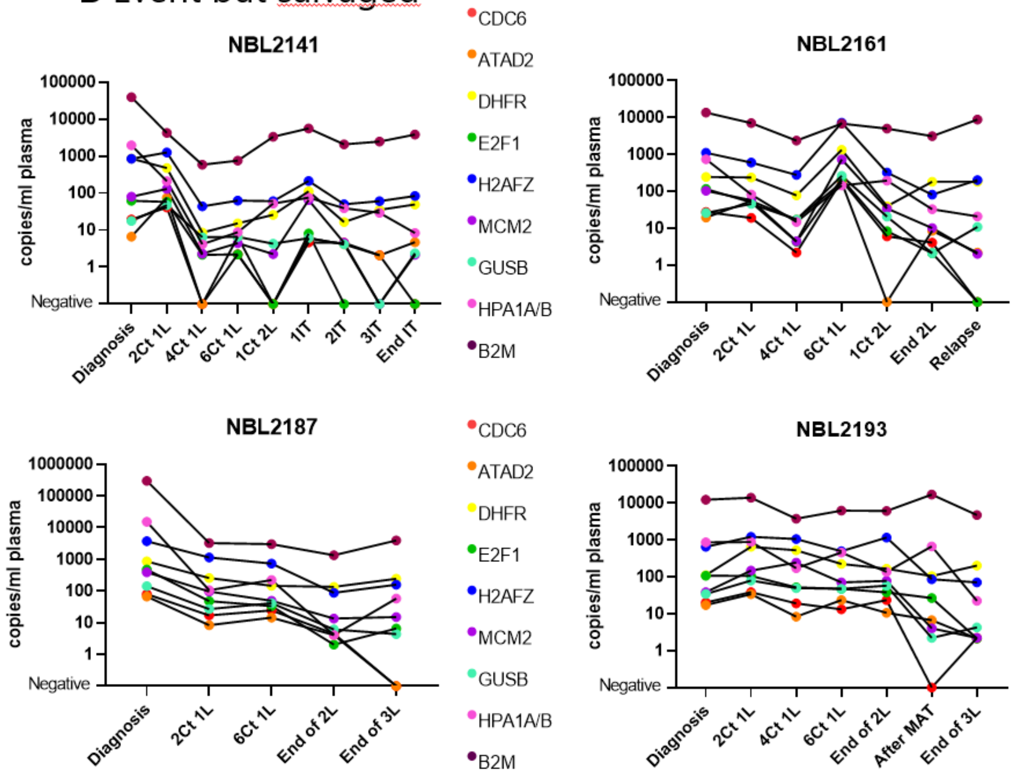

C Died of disease

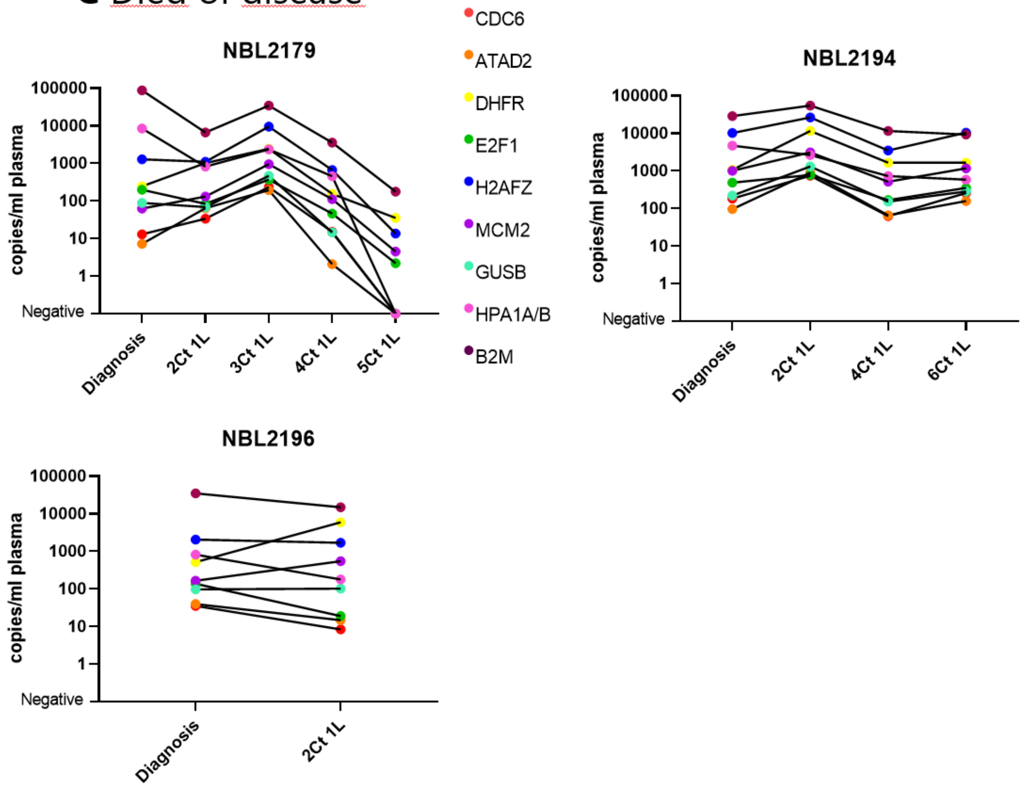

Supplemental Figure S4. Level of the cell cycle markers in sequential plasma samples from 11 patients with metastatic neuroblastoma. Patients are classified according to clinical outcome. **A.** No event (=no relapse/progressive disease and/or death of disease) **B.** Event but salvaged (=relapse/progressive disease but eventually complete remission or stable disease) **C.** Died of disease.

Dx; initial diagnosis
2Ct; 2nd chemotherapy course
4Ct; 4th chemotherapy course
1L; first line treatment
2L; second line treatment
End 3L; end of third line treatment
1IT; first course of immunotherapy
end IT; at the end of immunotherapy
After MAT; after myloablative therapy
R; relapse

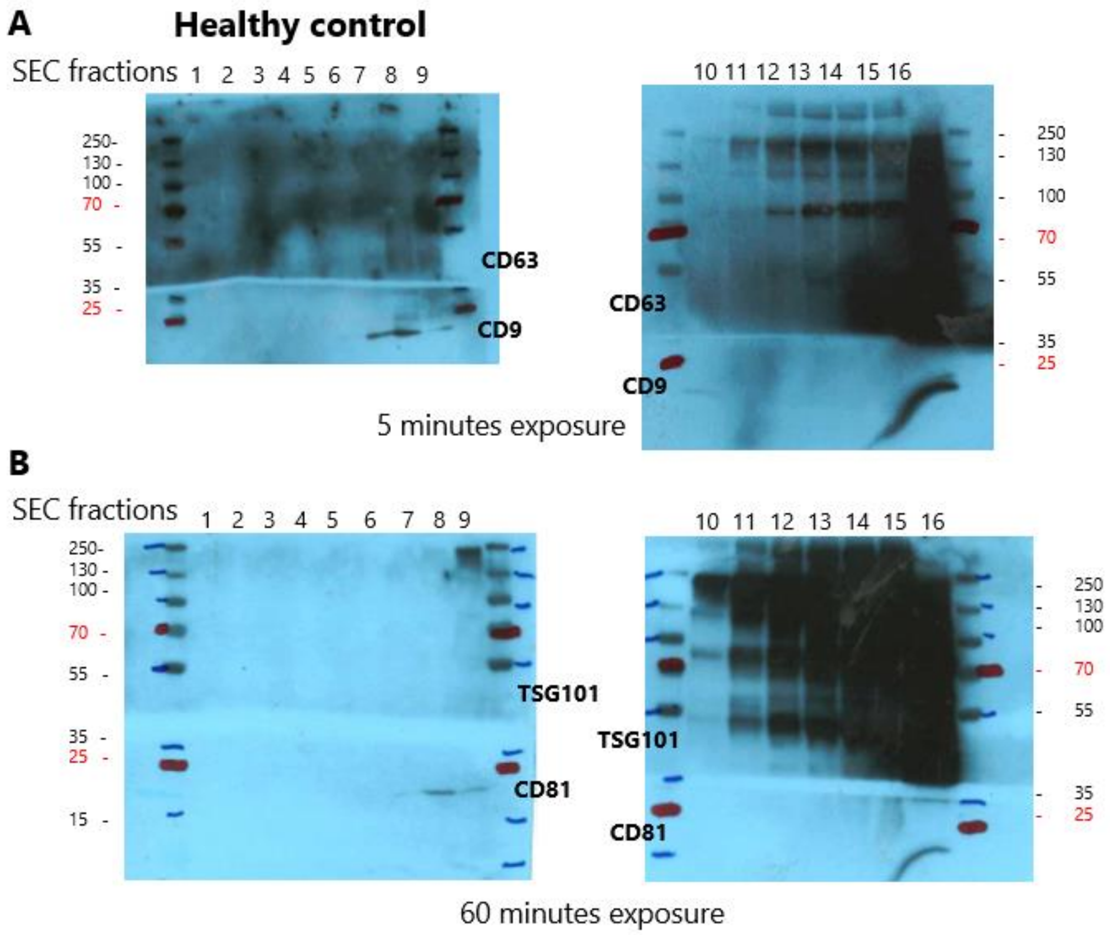

Supplemental Figure S5. Western blot images from the size exclusion chromatography (SEC) fractions isolated from 500ul of plasma from 1 healthy control. **A.** The blots were cut and first stained with CD63 (a smear around 44-55kDa) and CD9 (a band around 24kDa). SEC fractions 7, 8 and 9 are positive for CD9. CD63 is not clearly present in this blot, much aspecific staining in the higher protein-rich SEC fractions. **B.** Below, the same blots stained for TSG101 (band around 46kDa) and CD81 (band around 25kDa). SEC fractions 7, 8 and 9 are positive for CD81. SEC fractions 11 and further seem positive for TSG101 but much aspecific binding in these protein-enriched fractions.

Please note that protein input was not normalized in this experiment.

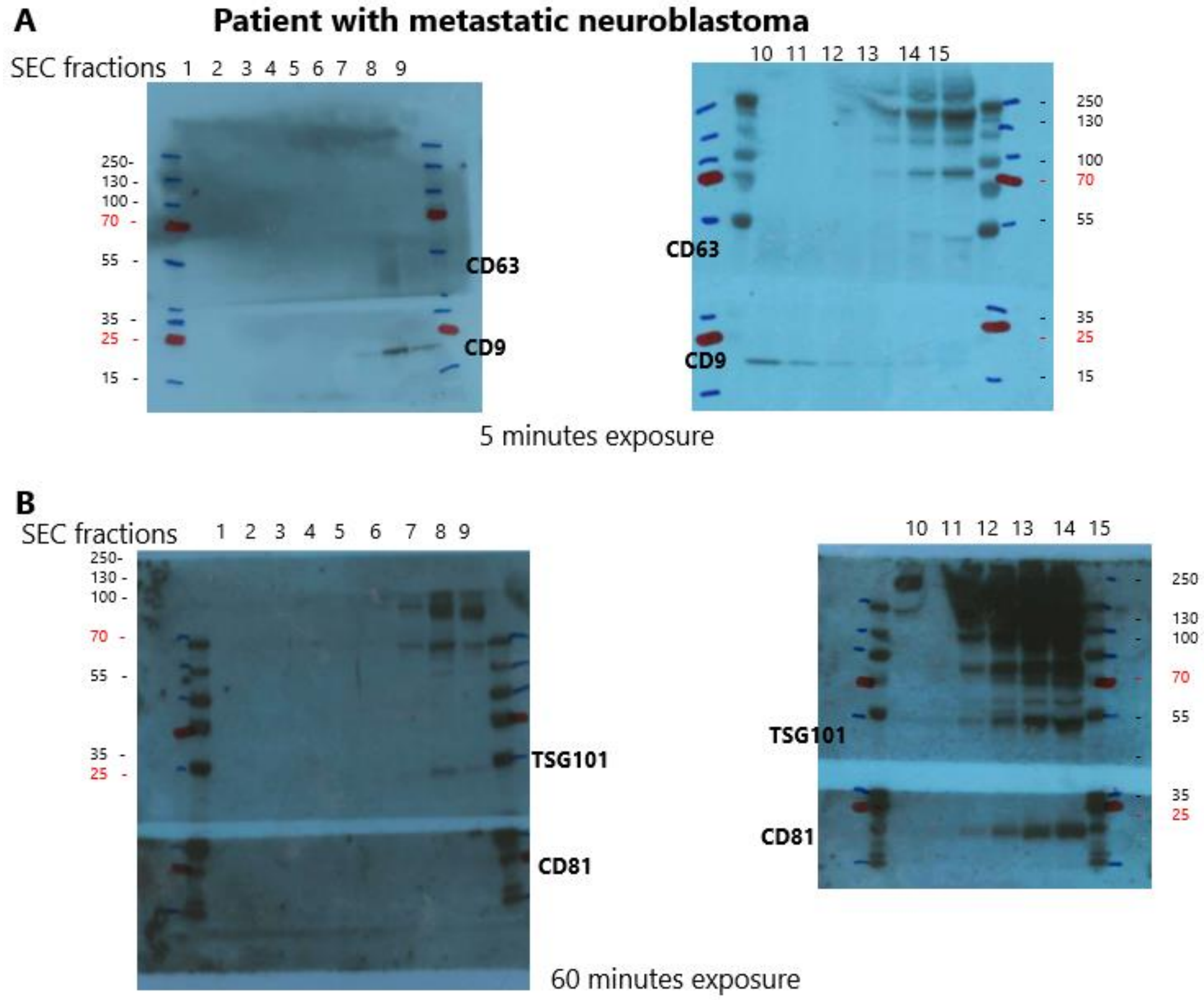

Supplemental Figure S6. Western blot images from the size exclusion chromatography (SEC) fractions isolated from 500ul of plasma from 1 patient with metastatic neuroblastoma. **A.** The blots were cut and first stained with CD63 (a smear around 44-55kDa) and CD9 (a band around 24kDa). SEC fractions 7, 8, 9, 10, 11 and 12 are positive for CD9. CD63 is present as a smear especially in SEC fraction 8, and some aspecific staining in the higher protein-rich SEC fractions. **B.** Below, the same blots stained for TSG101 (band around 46kDa) and CD81 (band around 25kDa). SEC fractions 12 and higher are positive for CD81. SEC fractions 7, 8 and 9, and later from 12 upwards are positive for TSG101 but some aspecific binding in the higher SEC fractions is present as well in the high molecular weight area.

Please note that protein input was not normalized in this experiment.

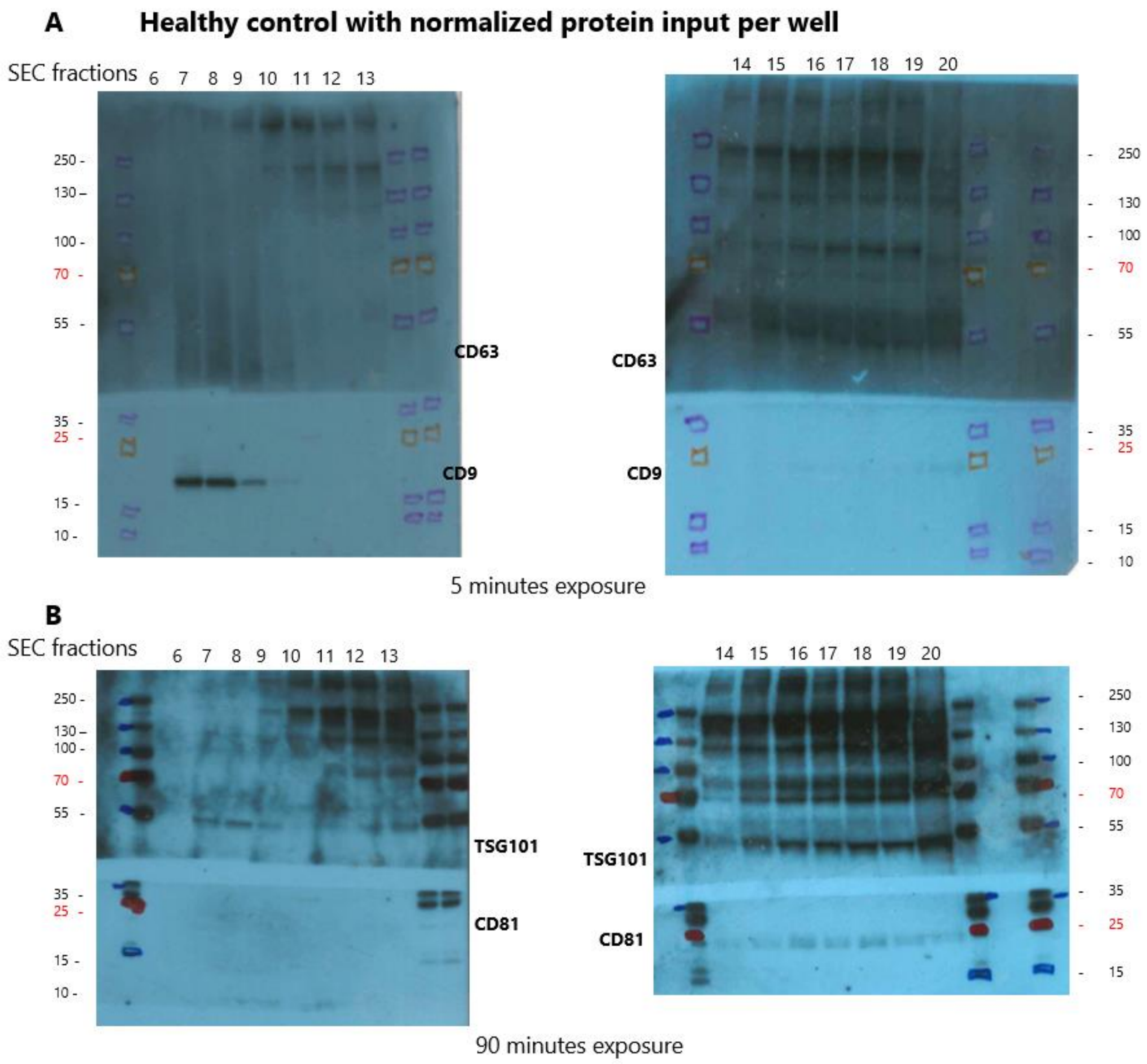

Supplemental Figure S7. Western blot images from the size exclusion chromatography (SEC) fractions isolated from 500ul of plasma from a healthy control. **A.** The blots were cut and first stained with CD63 (a smear around 44-55kDa) and CD9 (a band around 24kDa). SEC fractions 7, 8 and 9 are positive for CD9. CD63 is present as a smear in SEC fractions 7, 8 and 9 and faintly in 10. A smear might be present in SEC fractions 16 and upwards, with also some aspecific staining in the high molecular weight area. **B.** Below, the same blots stained for TSG101 (band around 46kDa) and CD81 (band around 25kDa). SEC fractions 16 and higher are positive for CD81. SEC fractions 7, 8 and 9, and later from 12 upwards are positive for TSG101 but some aspecific binding is present in the higher SEC fractions in the high molecular weight area as well.

Please note that protein input was normalized per well.

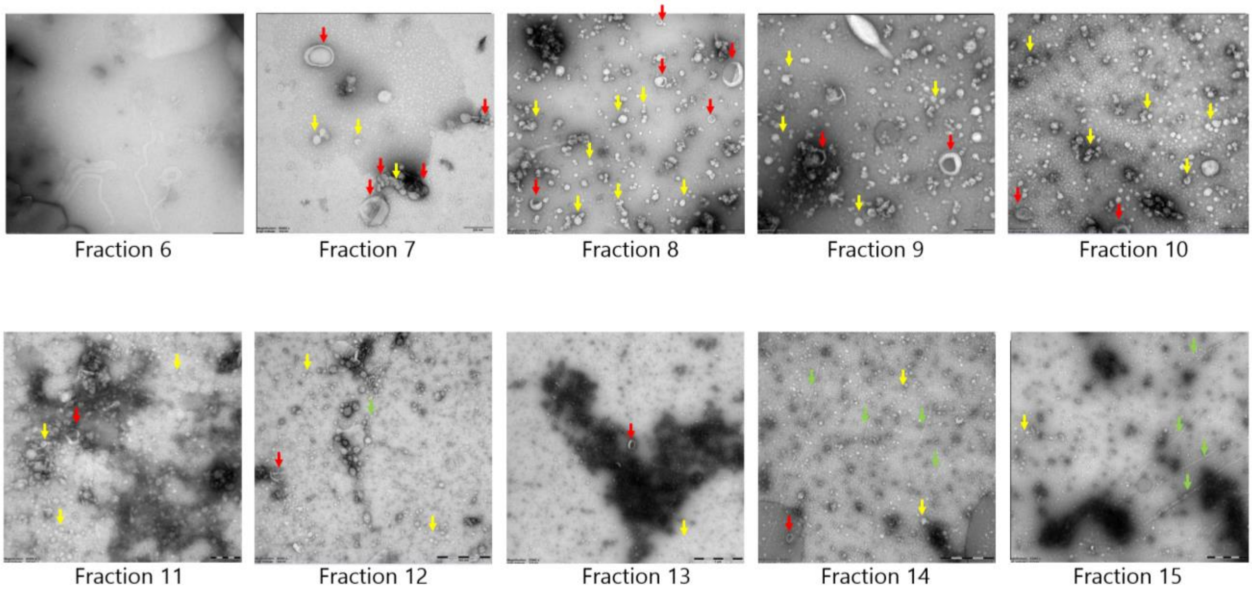

Supplemental Figure S8. Electron microscopy images of fractions (6 to 15) purified by size exclusion chromatography from 500ul of plasma from a patient with neuroblastoma. Red arrows indicate extracellular vesicles, yellow arrows lipoproteins and green arrows protein strands.

Supplemental Table S11. Results of the RNA-based ddPCR assays (both neuroblastoma-specific and cell cycle panels) and DNA-based ddPCR (*RASSF1A*-M and *ACTB*) on 200ul of SEC fractions isolated from 500ul of plasma.

		RNA													DNA	
		H2AFZ (copies/ml)	MCM2 (copies/ml)	CDC6 (copies/ml)	ATAD2 (copies/ml)	DHFR (copies/ml)	E2F1 (copies/ml)	GUSB (copies/ml)	B2M (copies/ml)	HPA1AB (copies/ml)	PHOX2B (copies/ml)	CHRNA3 (copies/ml)	TH (copies/ml)	GUSB nbl (copies/ml)	RASSF1A (copies/ml)	ACTB (copies/ml)
	**F6**	18.02074	0	0	0	2.21852	0	0	174.15	4.543044	0	0	0	0	0	264
	**F7**	214.2802	18.32361	0	0	42.70461	10.67615	8.556066	2120.087	54.51653	0	0	0	8.556066	0	748
	**F8**	121.905	20.97372	4.194744	2.210948	22.10948	22.10948	13.25055	1620.352	23.47239	2.150374	0	0	4.293177	352	2904
	**F9**	62.16399	6.428407	0	0	13.932	2.316952	4.633905	530.0218	11.35761	0	0	0	10.82759	0	264
	**F10**	23.09381	2.104944	0	0	12.56909	2.097372	0	212.0087	0	0	0	0	2.120087	352	4092
	**F11**	47.24766	6.163396	0	4.066024	81.77479	0	0	287.7261	6.057392	0	0	0	2.082229	792	7084
	**F12**	15.82494	2.256379	0	0	50.95781	0	2.210948	168.0926	15.14348	0	0	0	4.141742	132	968
**NBL2196**	**F13**	2.157946	2.157946	0	0	36.34435	4.543044	6.814566	67.38849	2.271522	0	0	0	2.0898	616	6072
	**F14**	12.41765	2.067085	0	2.014083	44.3704	0	0	120.3907	2.271522	0	0	0	0	0	5324
	**F15**	9.994697	0	0	0	96.1611	0	0	77.23175	0	2.04437	0	0	0	0	132
	**F16**	2.0898	0	0	0	18.17218	0	0	31.04413	2.271522	0	0	0	0	0	2200
	**F17**	2.029226	0	0	0	12.94768	2.157946	0	22.71522	0	0	0	0	0	0	8272
	**F18**	1.930794	0	0	0	6.481409	0	0	30.28696	0	0	0	0	2.036798	0	4576
	**F19**	0	2.014083	0	0	8.631784	0	0	17.415	4.543044	0	0	0	0	0	5324
	**F20**	0	0	0	0	14.31059	0	0	29.52979	0	0	0	0	2.150374	0	1012
	**F6**	28.99976	0	0	0	0	0	3.066555	393.7305	31.04413	0	0	0	4.573331	110	0
	**F7**	689.0283	85.56066	14.31059	19.08078	156.735	23.32096	36.04148	6579.842	408.874	0	0	0	25.59248	1188	3212
	**F8**	787.461	46.41477	8.101762	27.18255	159.0065	35.58718	29.30263	5678.805	401.3022	0	0	0	27.56113	2684	9284
	**F9**	264.2537	23.62383	7.87461	4.111455	109.0331	16.43068	12.34194	1567.35	106.7615	0	0	0	10.90331	3696	9020
	**F10**	123.4194	8.253197	0	0	156.735	10.75187	0	840.4631	60.57392	0	0	0	0	7876	25080
	**F11**	35.81433	8.404631	0	2.067085	49.5949	0	0	179.4502	8.328914	0	0	0	0	14520	48840
	**F12**	14.31059	0	0	0	113.5761	0	4.202316	124.9337	26.50109	0	0	0	0	24200	87120
**NBL2187**	**F13**	14.76489	4.209887	0	0	180.2074	0	0	74.20305	6.057392	0	0	0	0	24640	80960
	**F14**	8.253197	0	0	4.30832	276.3685	0	0	67.38849	6.814566	0	0	0	0	34320	100320
	**F15**	33.31566	1.961081	0	0	551.2227	0	0	65.87414	10.60044	0	0	0	0	8668	32120
	**F16**	8.101762	2.021655	0	0	320.2846	0	0	44.67327	9.086088	0	0	0	0	18920	65560
	**F17**	0	0	0	0	106.0044	0	0	25.74392	2.271522	0	0	0	0	33880	116160
	**F18**	8.404631	2.097372	0	0	135.5341	0	0	25.74392	4.543044	0	0	0	2.112515	32560	99000
	**F19**	10.82759	0	0	0	111.3046	0	0	12.11478	0	0	0	0	0	30360	121440
	**F20**	3.967592	0	0	0	69.50857	0	1.930794	8.328914	2.271522	0	0	0	0	26840	97240

Supplemental Table S12. Number of positive droplets per neuroblastoma-specific and cell cycle marker gene per 500ul SEC fraction, below the sum of all the droplets from the SEC fractions and as a comparison the number of droplets per marker from unfractionated 500ul plasma, of a healthy control and a neuroblastoma patient with metastatic disease.

		PHOX2B pos/drop /SECfr	CHRNA3 pos/drop /SECfr	TH pos/drop /SECfr	GUSB pos/drop /SECfr	CDC6 pos/drop /SECfr	ATAD2 pos/drop /SECfr	DHFR pos/drop /SECfr	E2F1 pos/drop /SECfr	H2AFZ pos/drop /SECfr	MCM2 pos/drop /SECfr	GUSB pos/drop /SECfr	HPA1A/B pos/drop /SECfr	B2M pos/drop /SECfr
**Control**	**F6**	0	0	0	8	0	3	1	1	41	2	1	NA	NA
	**F7**	0	0	0	15	1	1	19	5	282	19	12	1421	33337
	**F8**	0	0	0	8	0	0	13	2	149	12	12	327	10073
	**F9**	0	0	0	1	0	1	3	0	38	0	1	126	3875
	**F10**	0	0	0	0	3	0	2	2	25	1	1	88	2389
	**F11**	0	0	0	1	0	0	4	0	10	0	0	98	2217
	**F12**	0	0	0	0	0	0	1	0	11	3	0	99	2290
	**F13**	0	0	0	1	0	0	4	2	5	2	2	123	2137
	**F14**	0	0	0	0	0	0	1	0	5	0	0	86	1727
	**F15**	0	0	0	0	0	0	1	1	1	0	0	99	1441
	**F16**	0	0	0	1	0	0	1	0	4	0	1	84	1082
	**F17**	0	0	0	0	0	0	1	0	2	0	0	83	1012
	**F18**	0	0	0	0	0	0	6	3	1	0	0	91	1172
	**F19**	0	0	0	0	0	0	0	2	1	0	1	53	1016
	**F20**	0	0	0	0	0	0	3	0	1	1	0	59	712
**Total pos drop in all SECfr**		0	0	0	35	4	5	60	18	576	40	31	2837	64480
**Total pos drop in 500ul plasma**		0	0	0	86	17	38	197	182	1154	42	83	21346	42400
**NBL2177**	**F6**	0	0	0	1	0	0	6	5	16	0	0	49	338
	**F7**	4	13	0	23	5	6	33	51	470	50	17	612	5191
	**F8**	3	6	0	21	6	8	102	91	424	26	18	447	4783
	**F9**	0	5	0	7	2	1	140	35	161	17	8	161	1663
	**F10**	0	1	0	3	1	0	237	18	75	4	2	74	615
	**F11**	0	3	0	0	0	1	249	9	42	4	2	40	365
	**F12**	1	1	0	0	0	1	228	2	24	3	0	15	150
	**F13**	0	0	0	0	0	0	548	3	32	3	0	10	95
	**F14**	0	0	0	0	0	0	577	3	15	0	0	9	69
	**F15**	0	0	0	0	0	0	1358	0	41	2	0	6	58
	**F16**	0	0	0	0	0	1	729	2	25	2	1	9	85
	**F17**	0	0	0	3	0	0	232	0	14	0	0	5	63
	**F18**	0	0	0	0	0	0	186	0	11	1	0	3	49
	**F19**	0	0	0	0	0	0	191	0	7	0	0	9	43
	**F20**	0	0	0	0	0	0	261	0	6	3	0	2	36
**Total pos drop in all SECfr**		8	29	0	58	14	18	5077	219	1363	115	48	1451	13603
**Total pos drop in 500ul plasma**		13	10	0	27	21	23	9335	203	1511	69	30	1545	13739
